# Delayed Posterior Reversible Leukoencephalopathy Syndrome Triggered by FLOT Chemotherapy

**DOI:** 10.3389/fneur.2019.01405

**Published:** 2020-01-30

**Authors:** Jordi Gandini, Mario Manto, Nicolas Charette

**Affiliations:** ^1^Department of Neurology, CHU-Charleroi, Charleroi, Belgium; ^2^Service des Neurosciences, University of Mons, Mons, Belgium; ^3^Department of Gastroenterology, CHU-Charleroi, Charleroi, Belgium

**Keywords:** posterior reversible encephalopathy syndrome, reversible encephalopathy, sepsis, cancer, chemotherapy, sequelae

## Abstract

Posterior reversible encephalopathy syndrome (PRES) is a potentially severe disorder of the autoregulation of cerebral perfusion. The major clinical manifestations are headache, seizures, altered mental status, and visual loss. The typical radiological finding is vasogenic edema predominating in the white matter of occipital and parietal lobes. PRES is increasingly recognized as a clinico-radiological entity owing to improvements and fast availability of brain imaging, especially magnetic resonance imaging (MRI). We present the exceptional case of a 67-year-old female patient with a gastric adenocarcinoma at stage IIB (T3N0M0) treated by FLOT chemotherapy (5-fluorouracil, oxaliplatin, docetaxel, and folinic acid). Two months after the unique administration of FLOT regimen, she developed sudden posterior headache and visual loss. Blood pressure values were normal. Cerebral tomography showed ischemic-like occipital bilateral lesions, and angiographic sequences revealed breakdown of the blood–brain barrier (BBB). MRI revealed bilateral parieto-occipital T1 hypointensity and T2 hyperintensity, which demonstrated vasogenic edema. The rest of the parts of the lesions were T1 hyperintensity, T2 hyperintensity, and diffusion-weighted imaging (DWI) hyperintensity, which indicate cortical laminar necrosis. After injection of gadolinium, a linear enhancement of the cortex was observed. She was treated with oral nimodipine. Follow-up was characterized by permanent visual sequelae and tetraparesis. PRES represents an urgent neurological condition. Our observation highlights that PRES should be considered in patients under chemotherapy, even when their blood pressure remains within normal range. This is the first report of PRES triggered by FLOT chemotherapy and with a silent window of 2 months between chemotherapy and PRES, widening further the spectrum of chemotherapy-induced PRES. Our case highlights the potential role of FLOT regimen in the pathogenesis of PRES and the need for a novel approach in terms of prevention of this potentially fatal complication when patients receive chemotherapy.

## Introduction

Posterior reversible encephalopathy syndrome (PRES) is a clinico-radiological disorder of the autoregulation of cerebral perfusion, characterized by vasospasm of vertebrobasilar system (1–4). The main clinical manifestations include headache, seizures, altered mental status, and visual loss.

We report on a patient under chemotherapy who developed PRES despite normal blood pressure values and after a free interval of 2 months. We discuss our case in the light of the literature and emphasize the need to recognize this urgent neurological condition and develop novel approaches for prevention.

## Case Report

### Chief Complaints

A 69-year-old woman was admitted to the emergency room of our hospital for sudden headache with occipital topography, associated with nape pain and visual loss.

### Clinical Findings

She was under treatment by FLOT regimen (5-fluorouracil 4,200 mg, oxaliplatin 147.58 mg, docetaxel 87.5 mg, and folinic acid 350 mg) for a gastric adenocarcinoma at stage IIB (T3N0M0). The neoplasm infiltrated tunica serosa without lymph node infiltration or metastasis. The FLOT regimen was administered as a neoadjuvant treatment to prepare for the surgical procedure of removal of the lesion. She had received a unique dose of chemotherapy 2 months before admission. Chemotherapy was complicated by infectious pneumonia (*Streptococcus pneumoniae*) leading to septic shock, treated with intravenous infusion of amoxicillin/clavulanic acid, with acute renal failure requiring dialysis. For this reason, the chemotherapy was interrupted after administration. She had a personal history of arterial hypertension, vena cava and iliac deep vein thrombosis, polymyalgia rheumatica, hypercholesterolemia, chronic obstructive pulmonary disease (COPD), and blindness in the right eye. She was taking amiodarone, acetylsalicylic acid, tinzaparin, hydralazine, and lorazepam, but she was not taking any treatment for COPD.

### Diagnostic Assessment

On admission, blood pressure was 136/76 mmHg, weight 58.9 kg, height 1.63 m, heart rate 92 pulse/minute, body temperature 36.0°C, and capillary blood glucose 136 mg/dl. General physical examination was unremarkable. Neurological examination showed visual loss in the left eye and weakness of the lower limbs. Blood tests showed normal values of sodium and magnesium. Lactic acid dehydrogenase (LDH) levels were within normal limits. C-reactive protein (CRP) level was slightly increased, and albumin level was slightly decreased.

**Figure 1:**
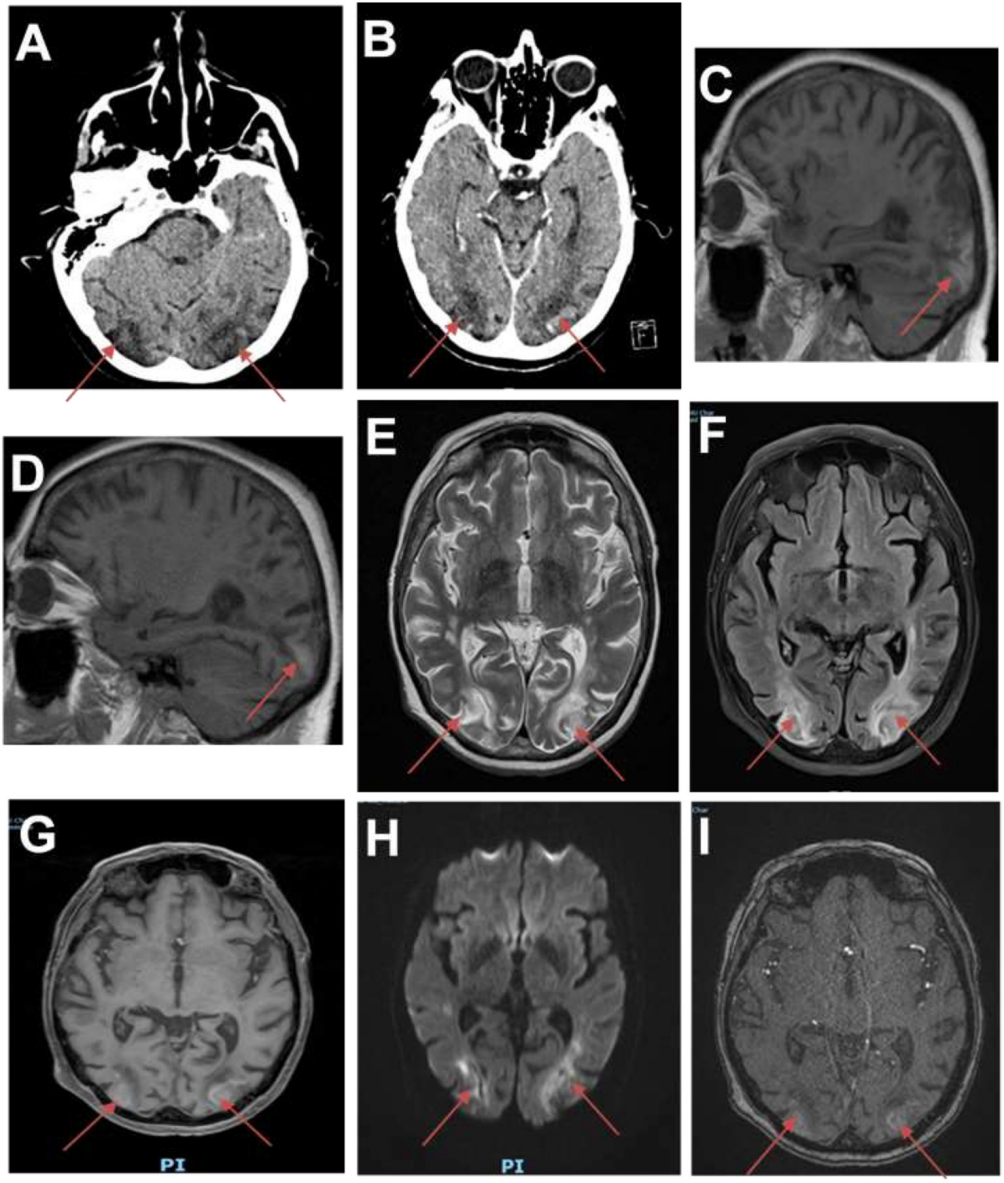
First brain imaging. **(A)** Brain CT showing ischemic-like lesions in the occipital lobes (red arrows). **(B)** CT scan showing hypodense lesions with breakdown of the blood–brain barrier (BBB) in the occipital lobes. **(C,D)** Sagittal T1 MRI showing hyperintensities in both occipital lobes. **(E)** Brain MRI (T2-weighted images) showing hyperintense lesions in the white matter of the occipital lobes. **(F)** Fluid attenuation inversion recovery (FLAIR) images showing ischemic-like lesions in the occipital lobes. **(G)** MRI angiographic sequences showing breakdown of BBB in the occipital lobes. **(H)** Diffusion sequence showing restriction in the occipital lobes. **(I)** Magnetic resonance angiography time-of-flight (MRA TOF) sequences showing breakdown of BBB in the occipital lobes.

**Figure 2:**
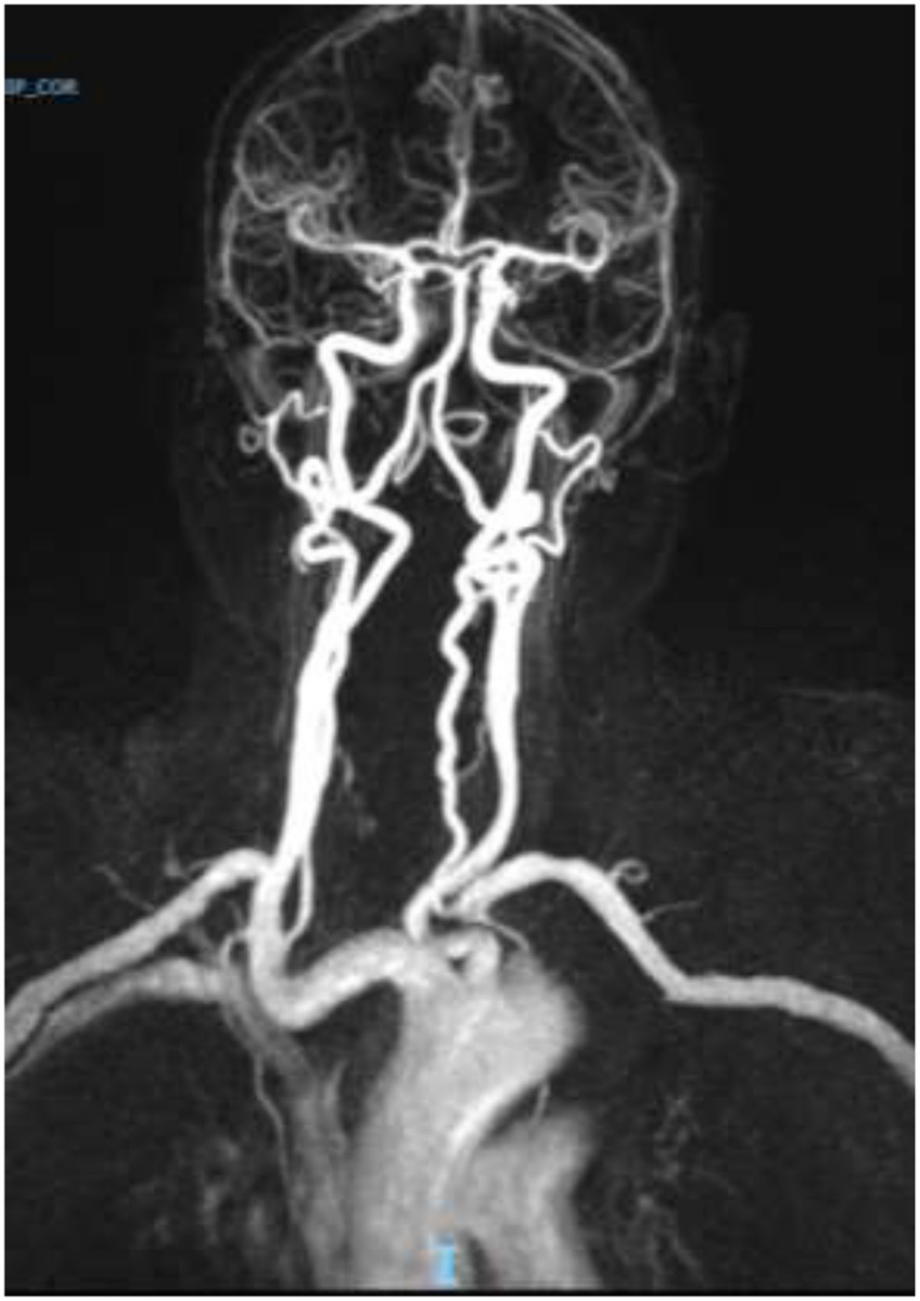
Normal magnetic resonance (MR) angiogram. No evidence of vasospasm in the anterior of the posterior circulation.

Brain computed tomography (CT) showed two ischemic-like occipital lesions without hemorrhage (Figure 1). Angiographic sequences revealed breakdown of the blood–brain barrier (BBB) in the corresponding regions. Cerebral magnetic resonance imaging (MRI) demonstrated bilateral parieto-occipital lesions: most parts of the lesions were T1 hypointensity and T2 hyperintensity, which demonstrated vasogenic edema. The rest of the parts of the lesions were T1 hyperintensity, T2 hyperintensity, and diffusion-weighted imaging (DWI) hyperintensity, which might indicate cortical laminar necrosis. After gadolinium injection, a linear enhancement of the cortex was observed. Multiple micro-ischemic lesions were observed in the periventricular regions, indicating a background of chronic ischemic leukoencephalopathy. No lesion was demonstrated in the posterior fossa. Dynamic susceptibility contrast (DSC)-MRI was not performed. MRI angiographic sequences did not show any abnormality in the vertebrobasilar system or in Willis polygon (Figure 2). In particular, there was no evidence of vasospasm.

Lumbar puncture was not performed in our patient owing to absence of clinical evidence of infectious meningitis. The electroencephalography (EEG) was unremarkable. The diagnosis of PRES was made, and the patient was admitted in our cerebrovascular unit to monitor her blood pressure and cardiorespiratory function. Regarding the blood pressure monitoring, she presented one single peak of hypertension (183/91 mmHg) a few hours after admission (see Supplementary Figure).

### Therapeutic Interventions

She was administered with oral nimodipine 360 mg/day because of the neuroprotective effect of this drug (5, 6). She left the hospital 48 h later with continuation of nimodipine at home and was followed up by ambulatory care as an outpatient.

### Follow-Up and Outcome

**Figure 3:**
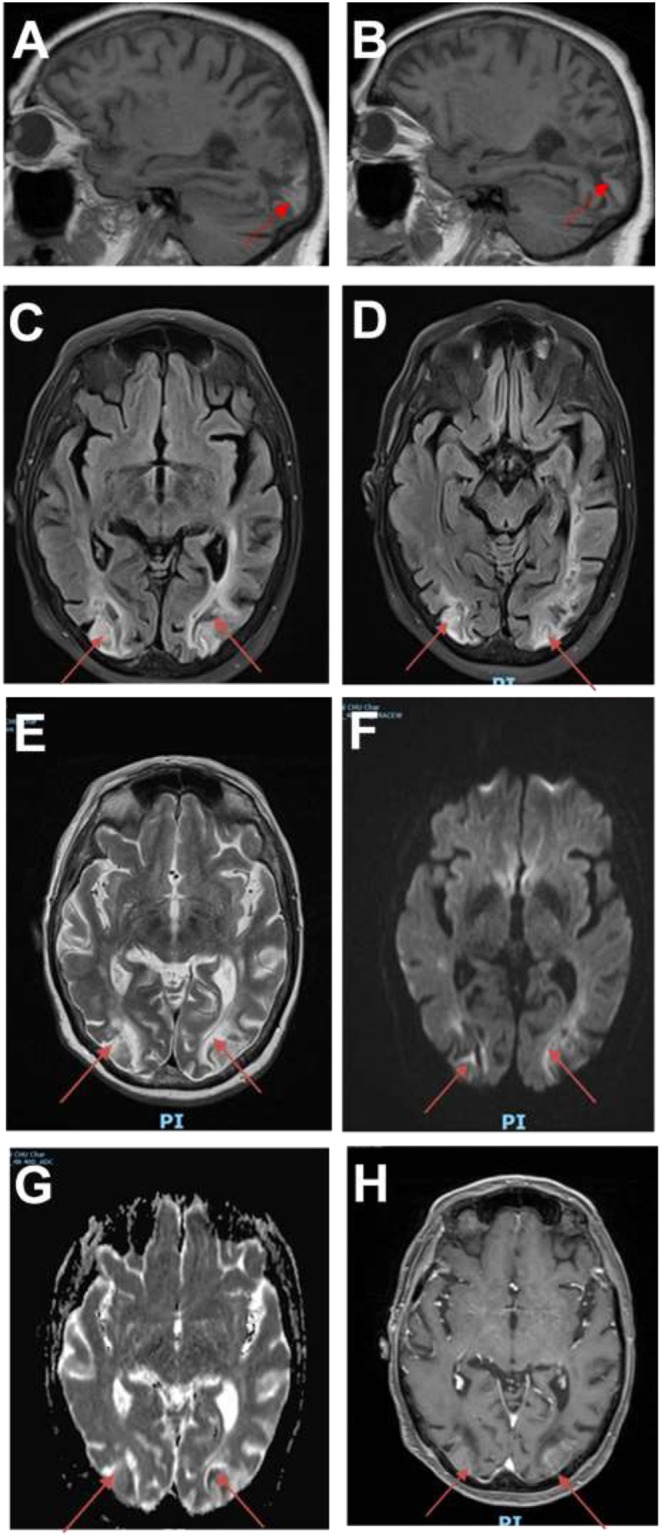
Follow-up images. **(A,B)** Sagittal T1 MRI showing hyperintensities in both occipital lobes (red arrows). **(C,D)** MRI fluid attenuation inversion recovery (FLAIR) images showing persistent ischemic-like lesions in the occipital lobes. **(E)** T2-weighted images showing hyperintense lesions in the occipital lobes. **(F)** Diffusion images demonstrating restriction in the occipital lobes. **(G)** MRI apparent diffusion coefficient (ADC) images showing residual restriction in the occipital lobes. **(H)** MRI angiographic sequences confirming persistent breakdown of BBB in the occipital lobes.

The clinical evolution was characterized by resolution of headache 1 month after discharge. The radiological follow-up with MRI 1 month later showed ischemic-like parieto-occipital bilateral lesions (Figure 3). There was no rechallenge with FLOT. The patient is alive 15 months after occurrence of PRES, with permanent visual sequelae and residual paraparesis.

## Pathophysiology

### The Theories to Explain Posterior Reversible Encephalopathy Syndrome

The pathophysiology of PRES remains controversial. At present, five theories have been proposed. First, the vasogenic theory postulates that severe hypertension causes interruption of cerebrovascular autoregulation (2, 3). Second, the endothelial theory considers that PRES is primarily due to an endothelial dysfunction caused by a systemic inflammatory state, triggered by toxics, sepsis, eclampsia, transplantation, or autoimmune disease (3). Third, the cytotoxic theory postulates that endothelial dysfunction is induced by endotoxins like chemokines or exotoxins like chemotherapeutic or immunosuppressive drugs (5). This theory can explain the occurrence of PRES during antineoplastic chemotherapy. Fourth, the immunogenic theory asserts that the first landmark of endothelial dysfunction is a T-cell-mediated inflammatory with chemokine release (5).

Given the limitations of the hypoperfusion/hyperperfusion theories above, Largeau et al. have postulated recently that PRES can be induced by hypersecretion of arginine vasopressin (AVP) or by an increase of AVP's receptor density. The authors have observed that PRES occurs in conditions with AVP hypersecretion such as sepsis or eclampsia. Activation of vasopressin V1a receptors would cause cerebral vasoconstriction, leading to endothelial dysfunction and cerebral ischemia. According to this novel theory, cytotoxic edema is induced by a transglial flux dysfunction with enhanced endothelial permeability, generating vasogenic brain edema. AVP can mediate also endothelial dysfunction through hypersecretion of vascular endothelial growth factor (VEGF) (6).

#### Chemotherapy and PRES: What Does Our Case Add to the Literature?

PRES occurs with certain imunosuppressive/chemotherapeutic drugs, monoclonal antibodies, and chemotherapeutic schemas (7–19). In 2016, How and colleagues reported 70 cases of PRES associated with chemotherapy. The most common chemotherapeutic agents were platinum salts (cisplatin, carboplatin, and oxaliplatin: 30 cases), daunorubicin (24 cases), vinca alkaloids (vincristine, vinorelbine, vinflunine, vinblastine, and vindesine: 21 cases), and 5-fluorouracil (13 cases); and only one case was associated with irinotecan (20). PRES has never been reported under chemotherapy with FLOT regimen, but one cannot exclude a key role of oxaliplatin in our patient, of course. FLOT is considered an efficient and safe neoadjuvant regimen for esophagogastric neoplasm (21, 22). Our article highlights the risk of PRES with this treatment so that oncologists can improve the monitoring of the risk factors in cancer. Our case expands the published reports on the associations of chemotherapies incriminated in PRES.

The pathophysiological relationship between chemotherapy and PRES remains to be further clarified. Several studies suggest that cytotoxic agents lead to endothelial dysfunction with production of vasoactive substances and trigger vascular leakage and edema development (23); this effect is amplified when several molecules are employed together (24). A study published by Liman and colleagues showed that patients with PRES who have received chemotherapy or immunosuppressive medication show significantly lower mean arterial pressure than did those with PRES from other etiologies (25).

Platinum salts are a well-known trigger of AVP hypersecretion (6), and they have a direct toxic effect on endothelial cells (26, 27).

5-Fluorouracil can cause a direct neurotoxic effect through the interruption of Krebs cycle or through thiamine deficiency (28, 29); a neurotoxic effect is reported also in case of dihydropyrimidine dehydrogenase (DPD), the initial and rate-limiting enzyme in the catabolism of fluoropyrimidines (30, 31).

#### Other Triggering Factors of Posterior Reversible Encephalopathy Syndrome: Relevance to Our Case

Concerning our patient, we have ruled out the other potential etiologies of PRES. She was treated with amoxicillin/clavulanic acid 2 months before admission, but only two antibiotics have been demonstrated to be associated with PRES: ciprofloxacin and linezolid (32, 33). In 2016, Van Aalst et al. described a case of PRES in a patient with an infected morphine pump (34). The patient was treated with intravenous amoxicillin/clavulanic acid, but she was withdrawn from opiates and underwent two surgical procedures to remove the infected pump. The second surgery was performed under general anesthesia; so she had at least three risk factors for PRES: infection, withdrawal (35), and general anesthesia (36). Therefore, amoxicillin/clavulanic acid cannot be considered an evident trigger factor of PRES at this stage.

Our patient suffered from polymyalgia rheumatica, but this disease is not associated with PRES (37). Furthermore, there is no evidence that PRES can be triggered by deep venous thrombosis of the leg or by vena cava thrombosis (37). Also, our patient suffered from COPD. β2-Agonists and corticosteroids employed in COPD are associated with PRES, but our patient did not take these drugs (37, 38). Amiodarone, acetylsalicylic acid, tinzaparin, hydralazine, and lorazepam are not associated with PRES (39). Septic shock and acute kidney insufficiency occurred 2 months before admission; in 2012, Kim et al. (40) described a case of PRES during a recovery from acute kidney injury (AKI), but the patient was still undergoing hemodialysis. We did not found any delayed case of PRES after AKI/dialysis.

### Clinico-Radiological Criteria: Criteria and Sequelae in Our Case

The first clinical signs of PRES are unusual headache and altered mental status (4, 41, 42). Nausea, vomiting, and seizures are described in 75% of patients. The epileptic crisis is initially focal and related to the topography of the lesional charge. A secondary generalization is common. Evolution into epileptic status may occur. Visual loss is present in more than 50% of the patients, but cortical blindness is rare. Some patients present weakness and loss of coordination of limbs (43).

In 2015, three clinico-radiological criteria were suggested for the diagnosis of PRES (44): neurological symptoms of acute onset, vasogenic edema on neuroimaging, and reversibility of clinical and/or radiological findings. Our patient met the first two criteria but showed visual sequelae and permanent paraparesis.

### Biological Findings

Serum findings are unspecific (38). Hypomagnesemia has been reported in the first 48 h of disease (45). Hypoalbuminemia has been observed in several patients with PRES of various etiologies (46, 47). LDH serum level has been proposed as a marker of endothelial dysfunction (48).

### Radiological Findings

Although angio-CT scan demonstrates posterior leukoencephalopathy and breakdown of BBB, MRI is the gold standard for the diagnosis of PRES (4, 49–51). The most common finding is edema without infarction of the sub-cortical white matter of the temporo-parieto-occipital lobes; this sign is usually bilateral and symmetric. Calcarine fissure and the paramedian area of occipital lobes are often spared (4, 41, 49). Gray matter is involved in only 30% of patients (50, 51). The involvement of the cerebellum, basal ganglia, internal capsule, frontal lobes, and brainstem is rare (52, 53). Four patterns of junctional distribution of lesions have been described: holo-hemispheric (23%), superior frontal sulcus (27%), primary parieto-occipital (22%), and “partial or asymmetric expression of the primary patterns” (28%) (38). The early phase of PRES is characterized by vasogenic edema, and the lesions are reversible. MRI may reveal hyperintensities in T2-weighted images and in fluid attenuation inversion recovery (FLAIR) sequences and isointense or hypointense lesions in T1-weighted images, whereas the diffusion sequences do not demonstrate abnormalities. Enhancement after gadolinium injection is described only in one third of cases (4, 42, 54, 55). By contrast, in the late phase of PRES, ischemic phenomena determine cytotoxic edema, and the lesions become irreversible. At this stage, T2-weighted images and FLAIR sequences demonstrate hyperintensities with or without microhemorrhages. Diffusion sequences reveal a low diffusion coefficient, which is the expression of ischemic lesions; this is correlated with irreversibility of this damage (56, 57). MRI can provide important information about the evolution of the disease: the reduction or the resolution of the abnormalities suggest the absence of ischemic lesions. By contrast, the persistence of the radiological anomalies indicates the ischemic installed lesions, similar to our case. In this situation, the involved areas may become atrophic with time (43). Spectroscopy can detect early perturbations of cellular metabolism in PRES, such as increase of lactic acid, creatine, and choline production or decrease of *N*-acetyl-aspartate (NAA) rate (51, 58). Scintigraphy and single-photon emission CT (SPECT) show hyperperfusion in the acute phase of PRES and hypoperfusion in the late phase (7).

### Differential Diagnosis

The most important differential diagnosis of PRES is ischemic stroke. In this case, the management of arterial hypertension is opposite. Several other diseases mimic the clinical presentation of PRES (59, 60).

MRI is critical for the differential diagnosis of the PRES. In particular, MRI allows a rapid diagnosis of ischemic stroke and cerebral thrombophlebitis. Black-blood angio-MRI may be helpful to make diagnosis of vasculitis: the typical pattern is characterized by thickening and enhancement of the vascular wall. A typical enhancement pattern was described for several diseases of the vessel wall (61–63). Cerebral angiography is often non-contributory, because the abnormalities of the vessel wall in case of vasculitis can determinate an angiographic pattern indistinguishable from that of PRES. Cerebro-spinal fluid (CSF) analysis is helpful in case of infectious or inflammatory diseases of the central nervous system (CNS); in this last category, blood cultures and serologic analyses are informative (43).

### Treatment

The first treatment of PRES is the control of trigger factors: suspension of immunosuppressive drugs or toxic agents, delivery in case of eclampsia, and correction of electrolytic or hemostatic disorders. The principal target of the treatment is the control of arterial hypertension; in particular, a mean arterial pressure between 105 and 125 mmHg is recommended. Cardiovascular monitoring is necessary in this early phase, and respiratory support is indicated if needed (4, 49). The antihypertensive therapy is based on three classes of molecules: calcium antagonists (nimodipine, nicardipine, and diltiazem), β-blockers (labetalol), and diuretics. The arterial dilators—sodium nitroprusside, diazoxide, and fenoldopam mesylate—are a second choice. Fenoldopam can induce a selective renal arteriolar dilatation with a beneficial effect in case of acute renal failure (64). Magnesium sulfate is proposed during pregnancy, due to a dilator effect on arterial wall, in particular in cerebral vessels (4, 43). Derived nitric oxide (NO) is not indicated, because it aggravates the cerebral edema (43). The invasive monitoring of arterial pressure is recommended in case of cardiac failure or precarious hemodynamic status (4).

The treatment of neurological complications is crucial: seizures are treated with benzodiazepines. When facing epileptic status, additional anti-epileptic drugs are required. For refractory epileptic status, deep sedation is indicated; thiopental, propofol, and midazolam are the gold standard. EEG monitoring is necessary for detection of the non-convulsive epileptic crisis and for verification of the efficiency of the therapy (4, 43).

### Prognosis

If the diagnosis is made quickly and if the patient is rapidly treated, clinical resolution often occurs within 7 days (42). The radiological resolution takes from 15 days to 1 year. In case of low apparent diffusion coefficient (ADC) at first MRI, the risk of irreversible lesions is increased. The prognosis is more severe, and a lethal outcome is possible for neoplastic diseases (43). Four predictive factors of fatal outcome have been identified: altered mental state, subarachnoid hemorrhage, raised CRP level, and altered coagulation (65). PRES is often accompanied by severe complications; neurological sequelae may persist (37).

## Conclusion

Clinical presentation of PRES is characterized by unusual headache and altered mental status, nausea, visual loss, vomiting, and seizures. Evolution to epileptic status is possible. Weakness and loss of coordination of limbs are rare. Arterial hypertension is frequent, but PRES has been reported in normotensive patients (66–69). MRI demonstrates edema without infarction of the sub-cortical white matter of bilateral temporo-parieto-occipital lobes. The involvement of the cerebellum, basal ganglia, internal capsule, frontal lobes, and brainstem is rare. Our case was unique by the silent interval between the unique dose of chemotherapy complicated by sepsis and the occurrence of the symptomatology of PRES.

Our patient presented multiple factors predisposing to breakdown of cerebrovascular regulation. We speculate that sepsis in an oncologic patient newly treated with platinum salts might have contributed to a cerebral dysregulation in the absence of arterial hypertension. However, because sepsis is a rather frequent complication in oncological immunosuppressed patients (70, 71), more studies are needed to confirm this hypothesis. The prevention of nosocomial infection, an appropriate vaccination before the administration of cytotoxic agents, and hygienic–dietary regimen could play a crucial role in the prevention of PRES in this category of patients who are especially exposed to this complication.

## Data Availability Statement

The datasets generated for this study are available on request to the corresponding author.

## Ethics Statement

Written, informed consent was obtained from the patient for the publication of this case report.

## Author Contributions

JG wrote the draft. MM and NC corrected the draft. All the authors approved the final version of the manuscript.

### Conflict of Interest

The authors declare that the research was conducted in the absence of any commercial or financial relationships that could be construed as a potential conflict of interest.
