# Supplementary material for: Delayed Posterior Reversible Leukoencephalopathy Syndrome Triggered by FLOT Chemotherapy
Source: Front Neurol. 2020 Jan 30;10:1405. doi: 10.3389/fneur.2019.01405 (PMC7002563; doi:10.3389/fneur.2019.01405)
Supplement: Supplementary file 1 [file Image_1.pdf]

Supplementary Material

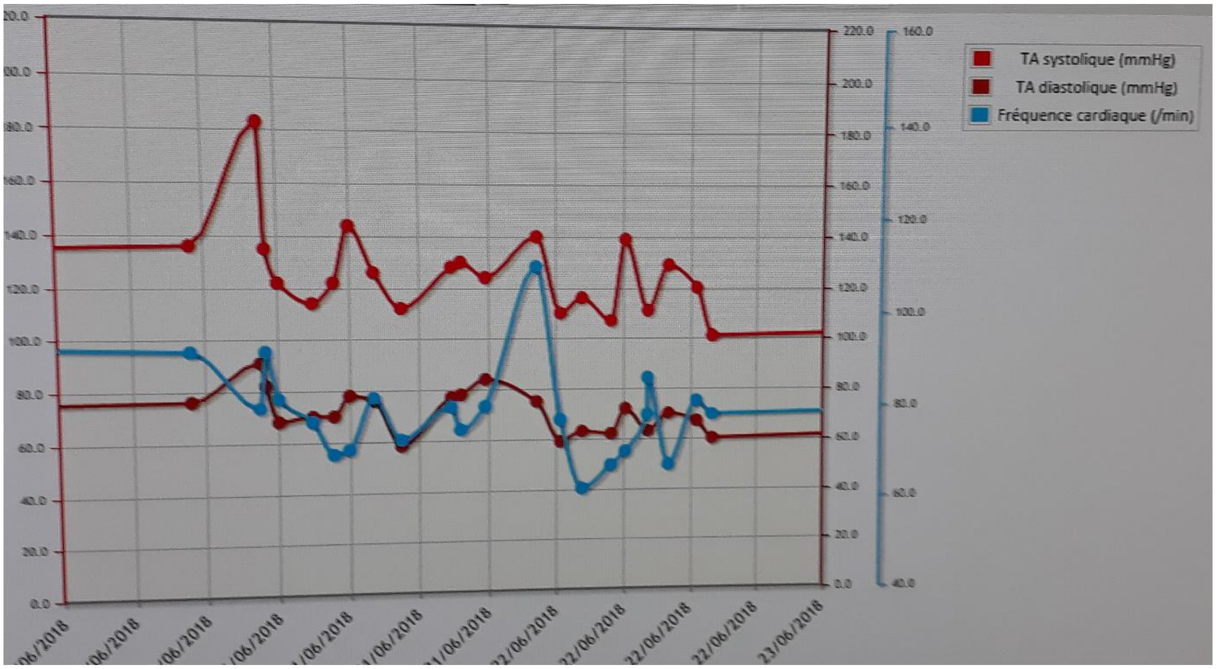

Supplementary Figure 1: Screenshot of the blood pressure monitoring in the neurovascular unit. Note the single peak of hypertension. Red lines: systolic (upper) and diastolic (lower) blood pressure values. Blue line: heart rate.
